# *In vitro* modulation of multidrug resistance by pregnane steroids and *in vivo* inhibition of tumour development by 7α-OBz-11α(R)-OTHP-5β-pregnanedione in K562/R7 and H295R cell xenografts

**DOI:** 10.1080/14756366.2019.1575825

**Published:** 2019-02-19

**Authors:** Ghina Alameh, Agnès Emptoz-Bonneton, Marc Rolland de Ravel, Eva L. Matera, Elisabeth Mappus, Patrick Balaguer, Luc Rocheblave, Thierry Lomberget, Charles Dumontet, Marc Le Borgne, Michel Pugeat, Catherine Grenot, Claude Y. Cuilleron

**Affiliations:** a ISPB-Faculté de Pharmacie, Université de Lyon, Université Lyon 1, Lyon, France;; b Fédération d’Endocrinologie du pôle Est, Hospices Civils de Lyon, Lyon, France;; c Centre de Recherche en Cancérologie de Lyon, Université Claude Bernard Lyon 1, INSERM, Centre Léon Bérard, Lyon, France;; d Institut de Recherche en Cancérologie de Montpellier, Université de Montpellier, Montpellier, France;; e Faculté de Pharmacie-ISPB, Department of Bioactive Molecules and Medicinal Chemistry, Université de Lyon, Université Claude Bernard Lyon 1, Lyon, France

**Keywords:** Multidrug resistance, P-glycoprotein, non-steroidogenic and steroidogenic cell lines, pregnane modulators, xenografts

## Abstract

Synthetic progesterone and 5α/β-pregnane-3,20-dione derivatives were evaluated as *in vitro* and *in vivo* modulators of multidrug-resistance (MDR) using two P-gp-expressing human cell lines, the non-steroidogenic K562/R7 erythroleukaemia cells and the steroidogenic NCI-H295R adrenocortical carcinoma cells, both resistant to doxorubicin. The maximal effect in both cell lines was observed for 7α-*O*-benzoyloxy,11α(R)-*O*-tetrahydropyranyloxy-5β-pregnane-3,20-dione **4**. This modulator co-injected with doxorubicin significantly decreased the tumour size and increased the survival time of immunodeficient mice xenografted with NCI-H295R or K562/R7 cells.

## Introduction

Reversion of multidrug resistance (MDR) remains an important goal to improve the efficiency of cancer chemotherapy. A major contributor to the MDR phenotype is P-glycoprotein (P-gp/ABCB1), a transmembrane protein belonging to the ABC (ATP-binding cassette) family of ATP-dependent transporters. This protein is often overexpressed in tumour cells after treatment by cytotoxic drugs and is also present in normal tissues such as blood-brain barrier, adrenal glands, kidney or liver, for which it has a physiological role to eliminate xenobiotics, toxics or drugs^1^. P-gp transports a wide variety of structurally unrelated molecules including most of the anticancer drugs, which undergo a mechanism of inactivation by drug efflux, at the origin of chemoresistance.

Different classes of molecules have been found able to inhibit P-gp-mediated drug efflux among which several pharmaceutical agents such as verapamil, diltiazem, nifedipine, cyclosporin A, valspodar^2^, or specifically designed P-gp modulators such as neratinib^3^, and numerous other compounds^4^. Comparative studies have revealed favourable structural features^5^ and provided data on the localisation of interaction sites with substrates or modulators^6^ including photoaffinity labelling^7^ and mutagenesis experiments^8^. However, various difficulties have been encountered in attempts to improve *in vivo* activities and to limit toxic side effects^9^ including unwanted pharmacokinetic interactions, intrinsic pharmacological activities as well as intrusion within the physiological role of P-gp in normal tissues, thus explaining why clinical trials have often led to unsatisfactory results.

**Figure 1.**
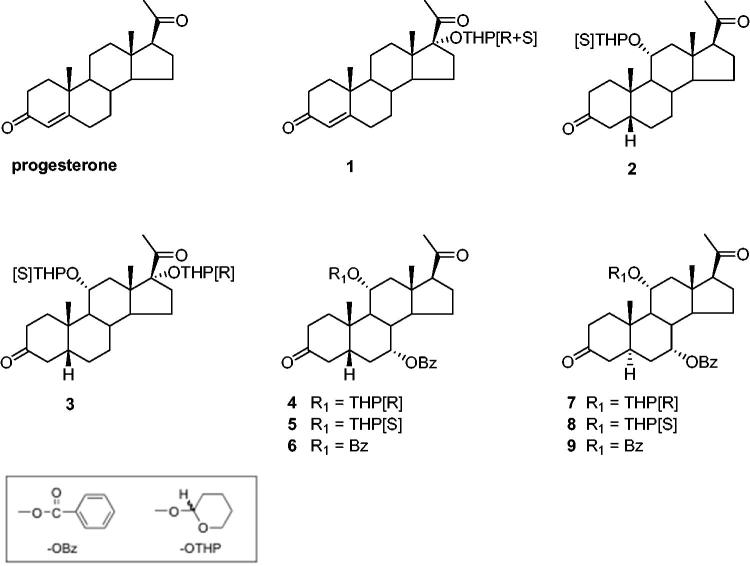
Structures of synthetic steroid modulators.

Since the initial observation of the P-gp-modulating properties of progesterone^10^, numerous studies have been undertaken to obtain improved modulators derived from steroidal hormones or drugs^11–14^. A major reason for this choice is the amount of data concerning their physiologic, pharmacologic, and metabolic properties as well as their toxicities, biological targets, and transport mechanisms. Moreover, steroids are multifunctional molecules facilitating structural modifications for the optimisation of pharmacological activities and for the control of metabolic degradation *in vivo*. Recently, our laboratory has proposed a simple methodology based on the conversion of hydroxylated precursors of 5α/β-pregnane and 5β-cholane series to ether and/or ester derivatives which provided efficient modulators of P-gp mediated MDR when tested *in vitro* on the resistant non-steroidogenic K562/R7 human erythroleukemia cell line^15^ using doxorubicin as cytotoxic drug (Figure 1).

In the present study, we report results of new biological investigations aimed at evaluating whether the nine progesterone and 5α/β-pregnane-3,20-dione MDR modulators **1–9** (Figure 1) previously selected using the K562/R7 cell line can maintain a high level of activity *in vivo*. In this view, three major obstacles could be expected for these compounds according to their structures: (i) the risks of metabolic deactivation by the enzymes of steroid metabolism, (ii) the potential hormonal side effects, (iii) the possibility of unacceptable levels of toxicity *in vivo*. These problems were successively addressed by comparative *in vitro* assays made on the human H295R adrenocortical carcinoma cell line rich in steroid metabolising enzymes and the metabolically inert K562/R7 cell line, by *in vitro* measurements of interactions with the human progesterone receptor (PR) and human pregnane X receptor (hPXR) and finally by *in vivo* assays made on mice xenografted with K562/R7 and NCI-H295R cell lines.

## Materials and methods

### Drugs and steroids

Doxorubicin, colchicine, vincristine, vinblastine, vinorelbine, paclitaxel, mitoxantrone, and cyclosporin A were purchased from Sigma. Syntheses of steroid modulators were previously described^15^. For *in vitro* experiments, dilutions in culture medium were extemporaneously made from 10 mM solutions in DMSO (or in water for cyclosporin A). For *in vivo* experiments, the steroid modulator **4** (2.7 mg) was sonicated in 100 µL of benzyl alcohol until solubilisation. This solution was diluted at 6% (v/v) in physiological serum before injections.

### Cell lines and culture conditions

The NCI-H295R human adrenocortical carcinoma cells (donated by M. Bégeot, INSERM-U864) previously characterised as expressing pathways of steroid biosynthesis^16^ were grown at 37 °C in a 5% CO_2_ atmosphere using a 1:1 mixture of DMEM and Ham’s F-12 medium, supplemented with L-glutamine (2 mM), antibiotics (50 µg/mL streptomycin, 50 U/mL penicillin), ITS + 1 (mixture of insulin, transferrin, and sodium selenite, from Sigma), 2% Ultroser G and SF (Life Science Technologies). The K562 human erythroleukemia cell line and the doxorubicin-resistant K562/R7 cell line (provided by C. Dumontet) were cultured as previously described^15^. The human promyelocytic HL60-MRP1 cell line overexpressing the Multidrug Resistance Protein 1 (MRP1/ABCC1) and resistant to doxorubicin and the human breast adenocarcinoma MCF7-MTX cell line expressing the Breast Cancer Resistance Protein (BCRP/ABCG2) and resistant to mitoxantrone (donated by A. di Pietro, IBCP-CNRS) were both cultured at 37 °C in a 5% CO_2_ atmosphere using either (HL60-MRP1 cells) an RPMI 1640 medium supplemented with 10% FCS, 58 nM of doxorubicin and 70 nM of vincristine or (MCF7-MTX cells) a 1:1 mixture of DMEM and Ham’s F-12 medium supplemented with 10% FCS, L-glutamine (2 mM), antibiotics (50 µg/mL streptomycin and 50 U/mL penicillin). Media and supplements were obtained from PAA (Velizy, France).

### Isolation of RNA and real-time RT-PCR

Total RNA extraction from NCI-H295R cells, first-strand cDNAs synthesis and real-time PCR were performed as previously described for K562/R7 cells^15^. Results were expressed as relative levels after normalisation by G3PDH (glyceraldehyde 3-phosphate dehydrogenase) mRNA.

### Cytotoxicity analysis by MTT assay

Viability of K562/R7 cells was determined by the 3–(4,5-dimethylthiazolyl-2)–2,5-diphenyltetrazolium bromide (MTT) reagent as described^15^^,^^17^.

### Cytotoxicity analysis by [^3^H]thymidine incorporation assay

NCI-H295R cells (150,000/400 µL of medium) were seeded into 24-well plates and after cell attachment (24 h), culture medium was replaced by fresh medium (1 ml) containing doxorubicin at 10 µM either alone or in presence of steroid modulators or cyclosporin A at three concentrations (0.1, 1, and 10 µM). After incubation for 24 h at 37 °C, the medium was replaced by medium containing 1 µCi/mL of [*methyl*-^3^H]thymidine] (GE-Healthcare). After another 24 h incubation, the medium was discarded and cells were precipitated by 1 ml of aqueous 10% trichloroacetic acid (TCA) solution. After incubation for 15 min at 4 °C, the precipitate was washed by 500 µL of 5% TCA, solubilised by addition of sodium deoxycholate (300 µL of 4% solution in 0.5 M NaOH)^18^ and counted for radioactivity (Tri-Carb 1900 CA, Packard). K562 and K562/R7 cells (150,000/400 µL of medium) were distributed in 24-well plates and incubated with 600 µL of medium containing doxorubicin, steroids or cyclosporin A as above, and treated by 1 µCi of [^3^H]thymidine as described for NCI-H295R cells. After treatment with TCA, cells were centrifuged (1000 rpm, 5 min), washed with phosphate-buffered saline (PBS), and counted for radioactivity. Results were expressed as the percentage of incorporated [^3^H]thymidine in treated cells vs. untreated cells. The assays were performed in triplicate and means ± SE were calculated from three independent measurements. For the determination of IC_50_ values, NCI-H295R or K562/R7 cells were incubated for 24 h with increasing concentrations of tested cytotoxic drugs (0.01–100 µM), in the absence or presence of a fixed concentration of steroid derivative and then treated with [^3^H]thymidine as described above. IC_50_ values were calculated with SigmaPlot-11.0.

### Progesterone receptor assay

The relative binding affinities of steroid modulators to PR were measured by a binding assay on a pool of breast tumour cytosols using [^3^H]ORG-2058 (Organon) as tracer^19^ (Experimental Section in SI).

### Activation of human pregnane X receptor by steroid derivatives

The activation of hPXR was evaluated using a luminescent assay^20^^,^^21^ (Experimental Section in SI).

### Flow cytometry

HL60-MRP1 and MCF7-MTX cells (10^−6^ cells) were incubated for 1 h at 37 °C in 1 ml of their respective culture medium containing 10^−5 ^M of either daunorubicin for HL60-MRP1 cells or mitoxantrone for MCF7-MTX cells, in the presence or absence of steroid modulators (10 µM)^22^.

### Animals

Female CB17-SCID (severe combined immunodeficient) mice (Charles River) were bred under pathogen-free conditions at the animal facility of our University. Animals were treated in accordance with the European Union and French laws for laboratory animal care after approval by the local animal ethical committee. The animals were kept in conventional housing. Access to food and water was unrestricted. All mice were 5/6 weeks-old at the time of tumour implantation.

### Xenografts of tumour cells and treatments of mice

K562 sensitive cells and K562/R7 or NCI-H295R resistant cells [3 × 10^6^ cells in 200 µL of a 50/50 mixture of PBS and Matrigel (Beckton-Dickinson)] were subcutaneously injected in both flanks of mice on day 1. Mice were then divided into four groups of 5 or 6 animals: group I received vehicle alone (100 µL/mouse of physiological serum containing 6% of benzyl alcohol); group II received simultaneously doxorubicin (1.5 mg/kg/mouse, dissolved in 50 µL of physiological serum) and vehicle (100 µL); group III received the steroid modulator **4** alone (8 mg/kg/mouse, dissolved in 100 µL of vehicle); group IV received simultaneously doxorubicin (1.5 mg/kg/mouse, dissolved in 50 µL of physiological serum) and the steroid modulator **4** (8 mg/kg/mouse, dissolved in 100 µL of vehicle) or cyclosporin A (10 mg/kg, dissolved in 100 µL of vehicle). This regimen corresponds to maximal doses for doxorubicin and modulator. For mice injected with NCI-H295R cells which developed a solid tumour 20 days later, treatments started on day 2 after cell injection. For K562/R7 cells which developed a solid tumour 40 days after injection of cells, treatment started on day 20 after injection. All treatments were ip administered every 4 days for 1 month. During treatment, mice were weighed and tumour progression was followed twice a week with an electronic caliper, using the formula: tumour volume (mm^3^)=length (mm)×width^2^/2. Animals were euthanized when tumour volume exceeded 3500 mm^3^ to avoid animal discomfort or if conditions suggested excessive suffering. Results were expressed as mean ± SE of tumour volumes in each group.

### Pharmacokinetic studies

Plasmatic concentrations of doxorubicin were determined after ip injections of doxorubicin (10 mg/kg dissolved in 50 µL of physiological serum) alone or in the presence of steroid modulator **4** (20 mg/kg, dissolved in 200 µL of vehicle) to three female OF1 mice per time point (0.5, 1, 2, 4, 8 and 24 h) (Experimental Section in SI).

### Statistical analyses

Data were expressed as means ± SE from a minimum of three independent experiments and were analysed by ANOVA followed by Student’s *t*-test. A *p* value <.05 was considered statistically significant.

## Results

### mRNA expression of ABC transporters in resistant NCI-H295R and K562/R7 tumour cell lines

RT-qPCR experiments made on NCI-H295R cells showed that P-gp/MDR1 mRNA was the major product (1525 copies, normalised by G3PDH) whereas very low amounts of mRNAs were expressed for the other ABC transporters MRP1 (ABCC1) (0.6 copy/G3PDH), BCRP (ABCG2) (0.2 copy/G3PDH) and MRP2 (ABCC2) (0.02 copy/G3PDH). These levels are much lower than reported for K562/R7 cells^15^ but remain in similar ratios. The presence of P-gp in the two NCI-H295R and K562/R7 cell lines was confirmed by Western blot using C219 anti-P-gp antibody (data not shown).

### Measurements of the cytotoxicity of doxorubicin in K562/R7 cells in the presence of steroid modulators using a [^3^H]thymidine assay

Cytotoxicity of doxorubicin in NCI-H295R and K562/R7 cells was measured by a [^3^H]thymidine incorporation assay less prone to steroid interferences than the MTT assay previously employed for non-steroidogenic K562/R7 cells^15^, owing to the risk of steroid interactions with the mitochondrial succinate dehydrogenase involved in MTT reduction^20^. In a preliminary experiment, K562/R7 cells were treated with doxorubicin alone and in the presence of progesterone or cyclosporin A (1 µM) as reference modulators of P-gp. Measurements with MTT or [^3^H]thymidine assays led to comparable IC_50_ values for doxorubicin. Similar measurements with the [^3^H]thymidine assay were made using a panel of four selected disubstituted 5α/β-pregnane modulators **3**, **4**, **6**, and **9** (Figure 1)^15^. The results (Table S1) showed that the 7α-OBz,11α(R)-OTHP-5β-pregnanedione lead compound **4** remained the most active modulator as found previously using the MTT assay^15^. However, many differences could be observed for the other steroid analogs owing possibly in part to the greater sensitivity of the thymidine assay. The intrinsic cytotoxicity of the four tested steroid modulators was estimated by treatment of nonresistant K562 cells for 24 h with a high steroid concentration (10 µM). The results (Table S1) showed that compounds **4** and **9** were less toxic than cyclosporin A as found previously using the MTT assay^15^.

**Table 1.** Modulation of resistance to doxorubicin by pregnane steroid derivatives.

Modulators (10 µM)		% [^3^H]thymidine incorporation (co-treated/untreated cells)^a^	
	Structure	H295R cells	K562/R7 cells
	pregn-4-ene-3,20-dione derivatives		
progesterone	17α-H	31.3±3.8	37.5±2.5
**1**	17α(R + S)-OTHP	11.6±1.9**	13.1±0.7**
	5β-pregnane-3,20-dione derivatives		
**2**	11α(S)-OTHP	25.0±1.6	19.3±0.2*
**3**	11α(S),17α(R)-diOTHP	12.6±2.8**	2.7±0.7**
**4**	7α-OBz,11α(R)-OTHP	3.6±0.9**	1.4±0.4**
**5**	7α-OBz,11α(S)-OTHP	13.4±3.1**	13.3±3.8**
**6**	7α,11α-diOBz	14.8±0.8**	5.7±0.3**
	5α-pregnane-3,20-dione derivatives		
**7**	7α-OBz,11α(R)-OTHP	19.0±2.2*	2.2±0.8**
**8**	7α-OBz,11α(S)-OTHP	13.5±1.3**	16.0±2.3*
**9**	7α,11α-diOBz	9.5±2.7**	6.1±2**
cyclosporin A	(non-steroidal modulator)	1.6±0.5**	2.2±0.3**

a Cells were treated with doxorubicin (10 μM) in the presence of steroid or cyclosporin A modulators (10 μM) for 24 h. Efficiency of modulators was evaluated through the strength of the decreasing effect on the percentage of [^3^H]thymidine incorporation in surviving co-treated cells vs. untreated cells. Significance was determined by ANOVA test, *p < .05 vs. progesterone, **p < .001 vs. progesterone.

### Resistance of NCI-H295R and K562/R7 cells to different classes of cytotoxic drugs

Different cytotoxic drugs (vinblastine, paclitaxel, mitoxantrone, colchicine, vinorelbine) were also tested on the two NCI-H295R and K562/R7 cell lines in comparison with doxorubicin (Table S2). Measurements by the [^3^H]thymidine assay after treatment for 24 h with varying concentrations of these drugs (0.01–100 µM) showed a strong resistance of NCI-H295R cells to doxorubicin reaching an IC_50_ value approximately one-half of that found for K562/R7 cells despite a much lower level of P-gp. The NCI-H295R cells also revealed a strong resistance to mitoxantrone (IC_50_ value 2-fold that of K562/R7 cells) but a low resistance to vinorelbine or paclitaxel and a very low resistance to vinblastine and colchicine. In contrast, K562/R7 cells, very resistant to doxorubicin, were found moderately resistant to mitoxantrone colchicine and vinblastine, but less resistant to vinorelbine and paclitaxel.

**Table 2.** Specificity of the interaction of steroids **3**,**4**,**6**,**9** with other ABC transporters.

Modulators (10 µM)	K562/R7 cells (P-gp)^a^	HL60-MRP1 cells (MRP1)^a^	MCF7-MTX cells (BCRP)^b^
**3**	2.5 ± 0.08	1.40 ± 0.10	1.80 ± 0.10
**4**	2.9 ± 0.15	1.35 ± 0.09	1.85 ± 0.09
**6**	2.6 ± 0.20	1.35 ± 0.12	1.70 ± 0.10
**9**	2.5 ± 0.10	1.70 ± 0.20	1.50 ± 0.15

a Cells were treated with 10 µM of daunorubicin (K562/R7 and HL60-MRP1 cells) in the presence or absence of 10 µM of steroid modulators and analysed by flow cytometry.

b Cells were treated with 10 μM of mitoxantrone in presence or absence of 10 µM of steroid modulators and analysed by flow cytometry. All the results are expressed as fluorescence ratio with/without modulator.

### Modulation of resistance to doxorubicin by different synthetic steroid derivatives in NCI-H295R and K562/R7cells

This study was undertaken to evaluate the modulating effects of nine steroid modulators **1**–**9** (Figure 1) selected from our previous *in vitro* study on K562/R7 cells^15^. Measurements were made on both NCI-H295R and K562/R7 cells, incubated for 24 h with doxorubicin (1 µM) in the absence or presence of steroid modulator (0.1, 1, and 10 µM). The results (Table 1) are expressed as the percentage of [^3^H]thymidine incorporated in treated cells vs. untreated cells. Treatment with doxorubicin alone led to a lower incorporation of [^3^H]thymidine in NCI-H295R cells (60%) than in K562/R7 cells (80%). In the presence of steroid modulators, the decrease in [^3^H]thymidine incorporation was roughly proportional to the steroid concentration. The efficiencies of the tested steroid derivatives were most often similar in the two cell lines, with exceptions for the dibenzoate **6** and the di-OTHP ethers **3** and **7**, both less active in NCI-H295R cells, owing possibly to partial metabolic conversion. In both cell lines, 7α-OBz,11α(R)-OTHP-5β-pregnane-3,20-dione **4** was the most efficient modulator in contrast to its 11α(S)-OTHP diastereoisomer **5** having a much lower efficiency. However, the efficiency of the steroid modulator **4** for NCI-H295R cells became lower than that of cyclosporin A, in contrast to the order observed for K562/R7 cells.

### Evaluation of interactions of pregnane modulators with human progesterone and pregnane X receptors

In order to evaluate the risks of unwanted side-effects, the four more potent 5α/β-pregnane modulators **3**, **4**, **6**, and **9** were tested for their ability to activate PR as well as hPXR owing to its role in the expression of ABC transporters and drug-metabolising enzymes^21^. None of the steroids at concentrations up to their respective IC_50_ values was able to significantly compete with the reference PR ligand [^3^H]ORG2058 (Figure S1(A)). Measurements of the activation of hPXR by a luminescent assay^23^ revealed different but significant effects of the four steroids. Activation increased between 0.1 and 1 µM concentrations of modulators (Figure S1(B)), but never reached the level induced by the reference non-steroidal hPXR activator SR12813. The lowest effect was observed for the steroid modulator **4** up to a 5 µM concentration.

### Specificity of the interaction of steroids with P-gp vs. other ABC transporters

The potency of the four steroid modulators **3**, **4**, **6**, or **9** to interact with MRP1 or BCRP was evaluated by measuring their modulating effects on the accumulation of daunorubicin in HL60-MRP1 cells or of mitoxantrone in MCF7-MTX cells (expressing BCRP), using flow cytometry. In HL60-MRP1 cells, the presence of 10 µM of steroid modulators led to a 1.3–1.7-fold increase of daunorubicin accumulation lower than the 2.5–2.9-fold increase found for P-gp-expressing K562/R7 cells^15^ (Table 2). In MCF7-MTX cells, the effects on mitoxantrone accumulation produced by the same steroids were much lower (2-fold increase) than that produced by elacridar, a reference modulator of BCRP (11-fold increase). Therefore, the four tested steroid modulators appeared to maintain a significant degree of specificity for P-gp in MDR inhibition assays *in vitro*. Interactions with MRP2, present only in trace amounts in K562/R7 or NCI-H295R cells, were not evaluated.

### *In vivo* efficiency of steroid modulator 4 in xenografted mice models

The *in vivo* activity of the steroid modulator **4** on MDR reversion was evaluated by monitoring tumour development in CB17-SCID mice xenografted with either NCI-H295R or K562/R7 cells. In preliminary experiments, the maximum tolerated dose of doxorubicin was evaluated to 1.5 mg/kg, whereas the dose of the steroid modulator **4** was limited to 8 mg/kg by its solubility.

For NCI-H295R cells, implantation of tumours in mice after sc injection was rapid (20 days) and treatments began 1 day after this injection. For K562/R7 cells, development of tumours in mice after sc injection was much slower (40 days) and therefore, treatments began 20 days after this injection.

**Figure 2.**
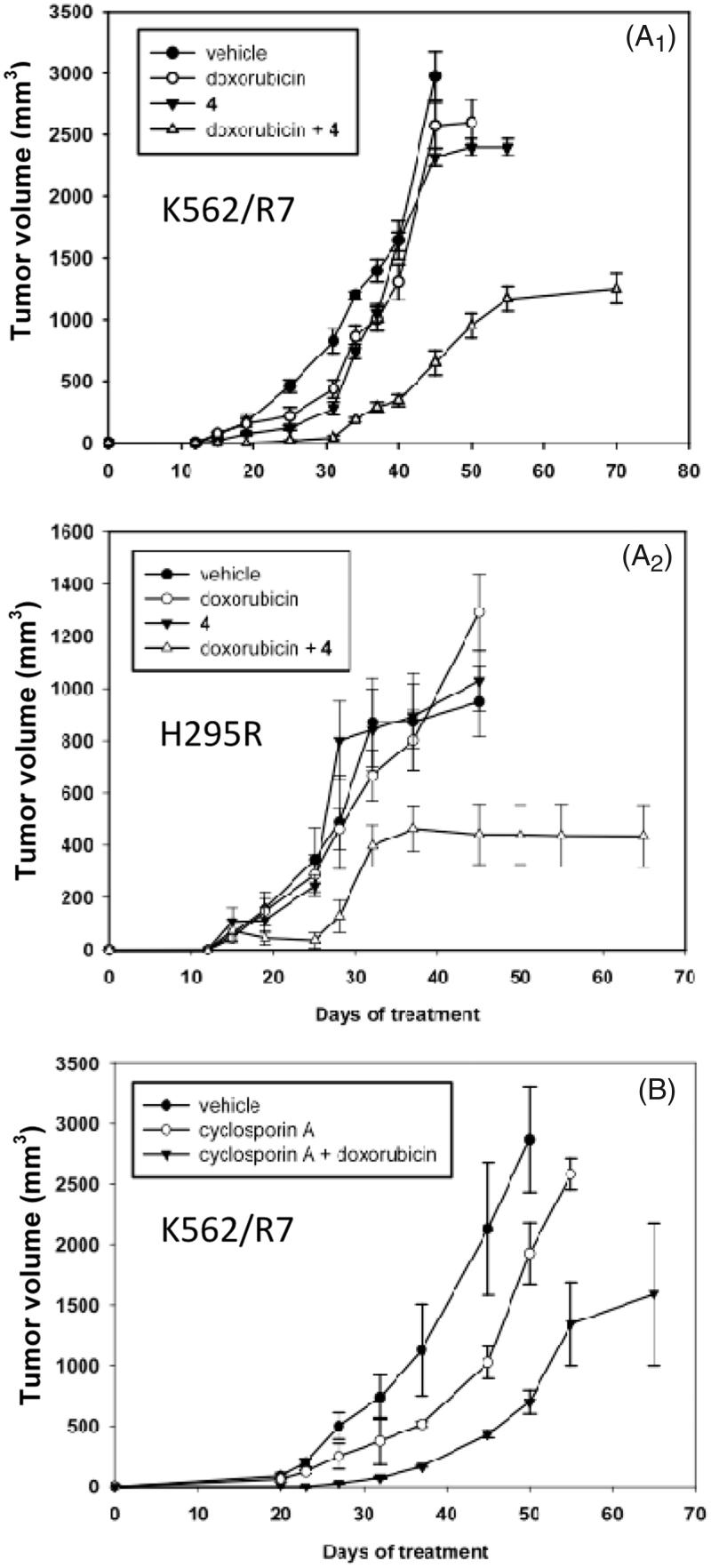
*In vivo* effect of co-treatment with doxorubicin + steroid modulator **4** or cyclosporin A on tumour volume of K562/R7 cell xenografts (panel A_1_) and H295R cell xenografts (panel A_2_). *In vivo* effect of co-treatment with doxorubicin + cyclosporine A on tumour volume of K562/R7 cell xenografts (panel B). Vehicle, doxorubicin, modulator **4** and doxorubicin + modulator **4** or cyclosporin A were administered ip. every 4 days during one month to groups of 6 mice as described in SI (Experimental Section). Results are expressed as mean ± SE.

In mice xenografted with K562/R7 cells, treatments with vehicle alone (group I), doxorubicin alone (group II) or steroid modulator **4** alone (group III) remained inefficient. The tumour volume reached either 500 mm^3^ (group I) or 200 mm^3^ (groups II and III) after the last injection (32 days) and then rapidly increased up to ca. 2600–3000 mm^3^ after 8–18 additional days without treatment (Figure 2(A_1_)). By contrast, co-treatment with doxorubicin and the steroid modulator **4** (group IV) prevented tumour development up to the last injection (32 days), then significantly limited tumour growth leading to a volume plateauing at ca. 1200 mm^3^, even after 38 further days without treatment. No apparent toxic effects were detected (no mouse died or presented any sign of morbidity). For mice xenografted with NCI-H295R cells, results (Figure 2(A_2_)) were similar to those observed for mice xenografted with K562/R7. Treatments with vehicle alone, doxorubicin, or steroid modulator **4** alone were inefficient, all leading to a tumour volume increasing up to ca. 1100 mm^3^ after 45 days including 13 days without treatment. In contrast, co-treatment with doxorubicin and the steroid modulator **4** reduced tumour development plateauing to 440 mm^3^ after 40 days including 8 days without treatment. No further increase of tumour volume occurred up to 25 additional days without treatment after which animals were sacrificed. No loss of weight or signs of morbidity were observed.

Conversely, co-administration of doxorubicin with the cyclosporin A modulator was found to be toxic (only 2 of 6 mice survived after the 4th injection – 16 days of treatment). This treatment was stopped to preserve the surviving mice for which the tumour volume (1500 mm^3^ after additional 49 days without treatment) was lower than in groups of untreated mice. On the other hand, treatment with cyclosporin A alone led to a tumour volume lower than in untreated mice (Figure 2(B)) suggesting an intrinsic toxicity of cyclosporin A.

For mice xenografted with sensitive K562 cells, treatment for 30 days with doxorubicin alone stabilised the tumour volume at ca. 2000 mm^3^, representing also nearly one-half of that reached without treatment (Figure S2).

### Effect of steroid modulator 4 on pharmacokinetics of doxorubicin

Several P-gp modulators have been reported to alter the pharmacokinetic properties of cytotoxic drugs^18^. The time course of plasma concentration of doxorubicin was measured in OF1 mice receiving a single ip dose of doxorubicin (4 mg/kg) in the absence and in the presence of steroid modulator **4** (20 mg/kg) at concentrations much higher than those tolerable by SCID mice. The results (Figure S3) showed no modifications of plasma concentrations of doxorubicin when co-administered with modulator **4**, indicating an absence of effect on the plasmatic half-life of doxorubicin.

## Discussion and conclusions

This work reports *in vitro* and *in vivo* data on K562/R7 and NCI-H295R cell lines that revealed the potency of the 7α-OBz,11α(R)-OTHP-5β-pregnane-3,20-dione lead compound **4**^15^ to modulate P-gp-mediated MDR and to inhibit the development of tumour cell xenografts. The K562/R7 cells, highly resistant to doxorubicin were found to express a much higher level of P-gp than the intrinsically resistant NCI-H295R cells that showed nevertheless half the resistance (IC_50_) to doxorubicin of that of K562/R7 cells (Table S1). This may suggest the presence of other non-elucidated factors than inhibition of P-gp efflux.

Comparisons of the modulating effects of steroid derivatives on doxorubicin resistance for the two NCI-H295R and K562/R7 cell lines as measured in this study either by the [^3^H]thymidine assay insensitive to potential steroid interferences in steroidogenic NCI-H295R cells or by the usual MTT assay^15^ revealed partially similar profiles. In all cases, the steroid modulator **4** showed the strongest modulating activity but the activities of compounds **3** and **7** were much lower for NCI-H295R cells (Table 1). None of the tested steroid modulators was found more active in NCI-H295R cells than in K562/R7 cells. The strongest activity of the steroid modulator **4** observed for both cell lines points to the conclusion that this steroid modulator is particularly resistant to metabolism in steroidogenic NCI-H295R cells that may provide a useful model for evaluating the risks of steroid metabolism *in vivo*. However, the stronger efficiency of steroid modulator **4** vs. cyclosporin A observed for K562/R7 cells was not maintained for H295R cells.

Neither the most efficient modulator **4** nor the three other less active steroids **3**, **6**, and **9** revealed any significant binding affinity for the progesterone receptor of human breast tumours. However, these four steroids were all found able to activate the hPXR of HG5N-PXR cells at the concentration of 0.4 µM (employed to reverse resistance to doxorubicin), among which compound **4** revealed the lowest effect. Activation of hPXR is known to increase chemoresistance via mechanisms involving overexpression of transport proteins and drug-metabolising enzymes^24^ and might become a major unwanted side-effect in the case of tumour cells expressing PXR, although beneficial effects have also been reported^25^. None of the tested derivatives could strongly reverse the resistance mediated by the two other ABC transport proteins MRP1 or BCRP, measured in HL60-MRP1 cells and in MCF7-MTX cells, respectively and present only in low amounts in the two studied K562/R7 or NCI-H295R cell lines.

Taken together, all these results point to the conclusion that compound **4**, the only potent steroid modulator found for NCI-H295R cells, was the most suitable candidate for undertaking *in vivo* assays.

*In vivo* experiments were performed on xenografts of K562/R7 or NCI-H295R cells introduced on SCID mice^26^. The maximum tolerated dose of doxorubicin (1.5 mg/kg) was limited by the extreme sensitivity of SCID mice to doxorubicin toxicity, owing to their deficit in cellular mechanisms of DNA repair^27^. The maximum dose of the 5β-pregnane modulator **4** (8 mg/kg) was imposed by its limit of solubility optimised by employing the lowest amount of benzyl alcohol (6%) able to maintain the steroid soluble in physiological serum without impairing mice survival. Non-xenografted SCID mice co-treated with these optimised doses of doxorubicin and steroid modulator could survive beyond 40 days, thus suggesting the absence of strong toxic side-effects of this treatment.

The potency of the steroid modulator **4** to inhibit tumour development *in vivo* was demonstrated in mice xenografted with either K562/R7 or NCI-H295R cells. In both cases, co-treatment with doxorubicin and modulator **4**, limited to 32 days in this study, reduced the tumour volume from 30–50% of that observed after treatments with only doxorubicin, steroid modulator or vehicle alone. These controls showed very weak effects on tumour development, thus confirming that xenografted cells remained highly resistant and suggesting the absence of intrinsic toxicity of the steroid. The co-treatment also increased the survival time up to 65–70 days without death cases. In contrast, control mice treated only either with doxorubicin or modulator **4** had to be sacrificed at 45–50 days, according to ethical rules, owing to excessive tumour size and poor health status. A complementary test made on mice xenografted with K562/R7 cells co-treated with doxorubicin and cyclosporin A showed a higher toxicity for mice and a lesser efficiency on tumour development as compared with co-treatment using the steroid modulator **4**. However, co-administration of doxorubicin and modulator **4** remained unable to block totally tumour development, owing possibly to the limited dose of doxorubicin that could be safely employed for treatments on SCID mice. This partial efficiency is in keeping with the results of parallel experiments made on SCID mice xenografted with sensitive K562 cells treated with doxorubicin alone.

On the other hand, compound **4** did not influence plasma pharmacokinetics of doxorubicin (tested on OF1 mice) thus supporting the assumption that the reversion of resistance is not caused by an increase of the plasma concentration of doxorubicin, but is more probably related to an inhibition of P-gp activity.

It should be noted that the choice of doxorubicin as cytotoxic agent in these comparative *in vitro* and *in vivo* evaluations of steroid modulators was made in the view to rely on a well-known drug despite its strong cardiotoxicity and its possible PXR-mediated stimulating effect on MDR1 expression^28^.

The results of this study are in agreement with similar *in vitro* and *in vivo* evaluations made on the human osteosarcoma MG63/DOX cell line overexpressing P-gp after co-treatment with doxorubicin and nilotinib^29^. The efficiency of the steroid modulator **4** supports the concept that potent MDR modulators can be obtained by simple esterification and/or etherification of hydroxylated steroid precursors. It remains to determine whether structural refinements of the substituents of steroid modulator **4** could reduce activation of hPXR and/or increase biodisponibility to further improve *in vivo* efficiency.

## Supplementary Material

Supplemental Material
