# Supplementary material for: In vitro modulation of multidrug resistance by pregnane steroids and in vivo inhibition of tumour development by 7α-OBz-11α(R)-OTHP-5β-pregnanedione in K562/R7 and H295R cell xenografts
Source: J Enzyme Inhib Med Chem. 2019 Feb 19;34(1):684–91. doi: 10.1080/14756366.2019.1575825 (PMC6383615; doi:10.1080/14756366.2019.1575825)
Supplement: Supplemental Material [file IENZ_A_1575825_SM6440.pdf]

Supporting Information

In vitro modulation of multidrug resistance by pregnane steroids and in vivo inhibition of tumor development by 7α-OBz-11α(R)-OTHP-5β-pregnanedione in K562/R7 and H295R cell xenografts

CONTENTS

Table S1. Modulation of IC_{50} values for doxorubicin by steroid inhibitors in K562/R7 cells and intrinsic toxicities of steroids in K562 cells.

Table S2. Resistance of H295R and K562/R7 cells to different cytotoxic drugs evaluated via their IC_{50} values

Figure S1. Hormonal activities of steroid modulators on PR and PXR.

Figure S2. *In vivo* activity of doxorubicin on tumor development in SCID mice xenografted with sensitive K562 cells.

Figure S3. Effect of steroid modulator 4 on pharmacokinetics of doxorubicin.

Experimental Section
- Progesterone receptor assay
- Activation of human pregnane X receptor by steroid derivatives
- Pharmacokinetic studies

Table S1. Modulation of IC_{50} values for doxorubicin by steroid inhibitors in K562/R7 cells and intrinsic toxicities of steroids in K562 cells.

modulators (concentration)	IC_{50} doxorubicin (µM)[a] (K562/R7 cells)	intrinsic toxicity (% surviving K562 cells)[b]
no modulator	23.6 ± 1.7	–
progesterone (3 µM)	14.9 ± 0.4	not determined
**3** (0.5 µM)	0.11 ± 0.01	67.3 ± 3.5
**4** (0.4 µM)	0.1 ± 0.007	81.5 ± 0.5
**6** (0.4 µM)	1.2 ± 0.06	68.2 ± 1.9
**9** (0.5 µM)	0.8 ± 0.05	80.0 ± 2.5
cyclosporin A (0.4 µM)	0.7 ± 0.01	69.2 ± 4.4

[a]IC_{50} values for doxorubicin were determined from dose-response curves obtained after incubation of cells for 24 h with different concentrations of doxorubicin (0.01 to 100 µM) in the absence or presence of a single concentration of steroid or cyclosporin A modulators. [^{3}H]thymidine incorporation was measured as described in Experimental section.
[b] Intrinsic toxicities of steroid or cyclosporin A modulators (10 µM) were determined in K562 sensitive cells after incubation for 24 h. [^{3}H]thymidine incorporation was measured as described in Experimental section.

Table S2. Resistance of H295R and K562/R7 cells to different cytotoxic drugs evaluated via their IC_{50} values.

cytotoxic drug	IC_{50} (µM)[a]	
	H295R cells	K562/R7 cells
doxorubicin	13.5 ± 2.9	23.6 ± 1.7
vinorelbine	1.3 ± 0.2	0.3 ± 1.3
taxol	0.4 ± 0.02	0.2 ± 0.04
vinblastine	0.07 ± 0.001	2.6 ± 0.2
mitoxantrone	10.0 ± 3.4	5.4 ± 1.9
colchicine	0.08 ± 0.001	3.5 ± 1.7

[a]IC_{50} values for cytotoxic drugs were determined from dose-response curves obtained after incubation of cells for 24 h with different concentrations of drugs (0.01 to 100 µM). [^{3}H]thymidine incorporation was measured as described in Experimental section.

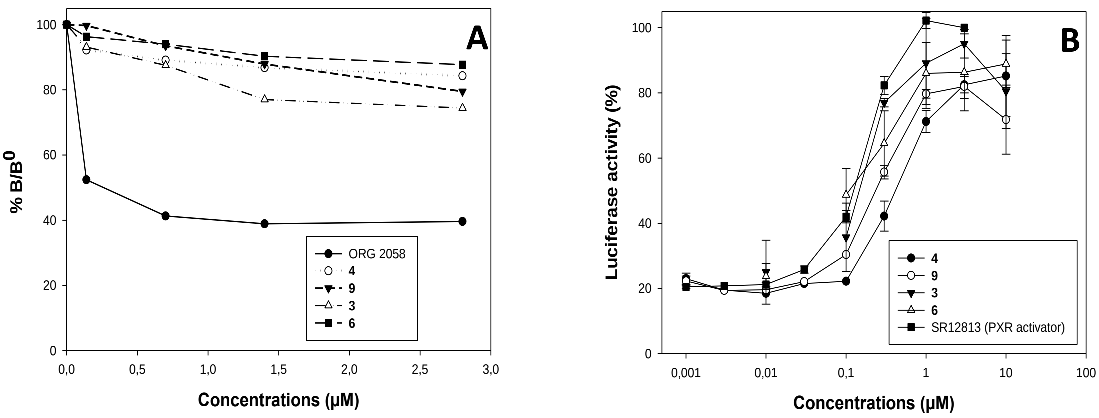

Figure S1. Hormonal activities of steroid modulators on PR and PXR.

Panel A: Relative binding affinities of steroid modulators for human progesterone receptor measured on a pool of breast tumors with the [^{3}H]ORG2058 ligand in absence or presence of unlabeled ORG2058 or steroid modulators as described in Experimental section. Results are expressed as the percentage of radioactivities B and B_0, respectively bound in the presence or absence of competitor.
Panel B: Activation of hPXR measured on HG_5N-PXR cells incubated with different concentrations (from 0.01 to 10 µM) of steroid modulators or of the reference hPXR activator SR12813 for 16 h. Luminescence was measured after addition of 0.3 mM of luciferin as described in Experimental Section. Results are expressed as the percentage of the value obtained using 3 µM of rifampicin activator.

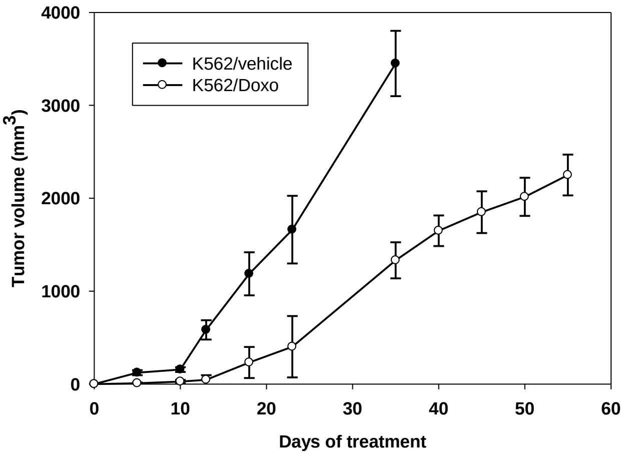

Figure S2. *In vivo* activity of doxorubicin on tumor development in SCID mice xenografted with sensitive K562 cells.
Vehicle or doxorubicin, were administered i.p. every 4 days during one month to groups of 6 mice as described in Experimental Section. Tumor development was followed by measuring tumor size twice a week with an electronic calliper. Results are expressed as mean ± SE.

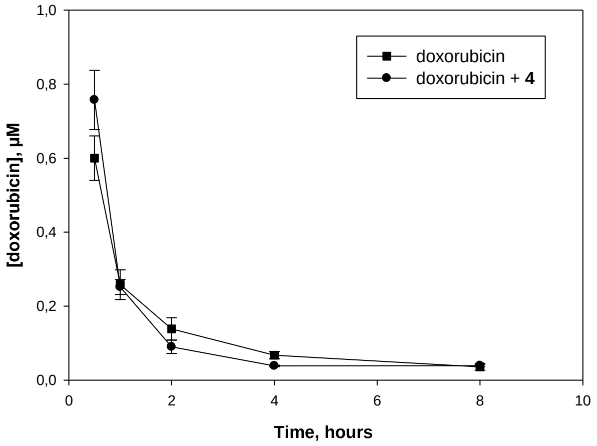

Figure S3. Effect of steroid modulator 4 on pharmacokinetics of doxorubicin.
OF1 mice were injected i.p. with doxorubicin alone or in presence of **4** and blood samples were collected at different times after the injection. Plasma concentrations of doxorubicin at each time were measured as described in Experimental Section.

Experimental Section

Progesterone receptor assay

The relative binding affinities of steroid modulators to progesterone receptors (PRs) were measured by a dextran-coated charcoal (DCC)-competitive binding assay[20]. Aliquots (80 µL) of cytosol (from a pool of breast tumors expressing high levels of PRs) were incubated overnight at 4 °C in microtitration plates with 20 µL of the PR ligand [^{3}H]ORG-2058 (Organon) (10 000 cpm, 1 nM), in absence or presence of unlabeled competitors (10, 50, 100, 200 nM). Free and bound steroids were separated following incubation for 15 min at 4 °C with 100 µL of DCC suspension (0.125% activated charcoal, 0.0125% dextran, in 10 mM Tris-HCl, pH 7.4) and centrifugation at 2200 rpm for 15 min.

Activation of human pregnane X receptor by steroid derivatives

The activation of human pregnane X receptor (hPXR) was evaluated using reporter HG_5LN-PXR cells containing a luciferase reporter gene under the control of a GAL4-yeast transcription factor-binding site stimulated by activation of the hPXR ligand-binding domain fused to the GAL4 DNA-binding domain (30, 31). HG_5LN-PXR cells and untransfected HG_5LN control cells were seeded (5×10^4 cells/well) in 96-well white opaque tissue culture plates (Becton-Dickinson) and grown in 200 µL of DMEM without phenol red but supplemented with 6% of steroid-free foetal calf serum (stripped with DCC). Different concentrations of steroids (0.01 to 10 µM) or of the reference hPXR activator SR12813 (0.01 to 5 µM) were added 24 h later and incubated for 16 h. The incubation medium was replaced by culture medium containing 0.3 mM luciferin then luminescence of intact living cells was measured for 2 s (MicroBeta-Wallac luminometer) and expressed as relative luminescence units (RLU). The results were expressed as a percentage of the value measured with the hPXR activator, rifampicin (3 µM). The experiments were performed in quadriplicate and means ± SD were calculated from three independent experiments.

Pharmacokinetic studies

Groups of three female OF1 mice per time point were i.p. injected with doxorubicin (10 mg/kg dissolved in 50 µL of physiological serum) alone or in the presence of steroid modulator **4** (20 mg/kg, dissolved in 200 µL of vehicle).

Blood samples were collected by puncture into the retroorbital vein after 0.5, 1, 2, 4, 8 and 24 h. After centrifugation, the plasma samples were stored frozen at -20 °C until analysis. Doxorubicin was isolated from 100 µL of plasma by precipitation of proteins with 200 µL of acetonitrile. After centrifugation for 6.5 min at 13000 rpm, the presence of doxorubicin in the supernatant was analyzed by HPLC using a Nucleosil C18 reverse-phase column (4.5 × 150 mm, Macherey-Nagel) equilibrated with a 80/20 mixture of 0.1% aqueous trifluoroacetic acid (TFA) and acetonitrile containing 0.06% TFA. Doxorubicin was eluted at 12 min by a gradient of aqueous acetonitrile (20% to 100% in 40 min). Peaks of doxorubicin were detected at 480 nm. The concentrations of doxorubicin were calculated by integration of the HPLC peaks using a standard curve.
